# 11β-Hydroxysteroid dehydrogenase type 1 contributes to the balance between 7-keto- and 7-hydroxy-oxysterols *in vivo*

**DOI:** 10.1016/j.bcp.2013.02.002

**Published:** 2013-07-01

**Authors:** Tijana Mitić, Steven Shave, Nina Semjonous, Iain McNae, Diego F. Cobice, Gareth G. Lavery, Scott P. Webster, Patrick W.F. Hadoke, Brian R. Walker, Ruth Andrew

**Affiliations:** a Endocrinology, University/British Heart Foundation Centre for Cardiovascular Science, The Queen's Medical Research Institute, University of Edinburgh, Edinburgh, EH16 4TJ, UK; b Institute of Structural and Molecular Biology, King's Buildings, University of Edinburgh, Edinburgh, EH9 3JR, UK; c Centre for Endocrinology, Diabetes and Metabolism, School of Clinical & Experimental Medicine, University of Birmingham, Birmingham, B15 2TT, UK

**Keywords:** 7-Oxysterols, 7β-Hydroxycholesterol, 7-Ketocholesterol, Glucocorticoids, 11β-Hydroxysteroid dehydrogenase 1, Corticosterone

## Abstract

11β-Hydroxysteroid dehydrogenase 1 (11βHSD1; EC 1.1.1.146) generates active glucocorticoids from inert 11-keto metabolites. However, it can also metabolize alternative substrates, including 7β-hydroxy- and 7-keto-cholesterol (7βOHC, 7KC). This has been demonstrated *in vitro* but its consequences *in vivo* are uncertain. We used genetically modified mice to investigate the contribution of 11βHSD1 to the balance of circulating levels of 7KC and 7βOHC *in vivo*, and dissected *in vitro* the kinetics of the interactions between oxysterols and glucocorticoids for metabolism by the mouse enzyme.

Circulating levels of 7KC and 7βOHC in mice were 91.3 ± 22.3 and 22.6 ± 5.7 nM respectively, increasing to 1240 ± 220 and 406 ± 39 nM in *ApoE*^*−/−*^ mice receiving atherogenic western diet. Disruption of 11βHSD1 in mice increased (*p* < 0.05) the 7KC/7βOHC ratio in plasma (by 20%) and also in isolated microsomes (2 fold). The 7KC/7βOHC ratio was similarly increased when NADPH generation was restricted by disruption of hexose-6-phosphate dehydrogenase.

Reduction and oxidation of 7-oxysterols by murine 11βHSD1 proceeded more slowly and substrate affinity was lower than for glucocorticoids. *in vitro* 7βOHC was a competitive inhibitor of oxidation of corticosterone (*K*_*i*_ = 0.9 μM), whereas 7KC only weakly inhibited reduction of 11-dehydrocorticosterone. However, supplementation of 7-oxysterols in cultured cells, secondary to cholesterol loading, preferentially slowed reduction of glucocorticoids, rather than oxidation.

Thus, in mouse, 11βHSD1 influenced the abundance and balance of circulating and tissue levels of 7βOHC and 7KC, promoting reduction of 7KC. In health, 7-oxysterols are unlikely to regulate glucocorticoid metabolism. However, in hyperlipidaemia, 7-oxysterols may inhibit glucocorticoid metabolism and modulate signaling through corticosteroid receptors.

## Graphical abstract

**Figure:**
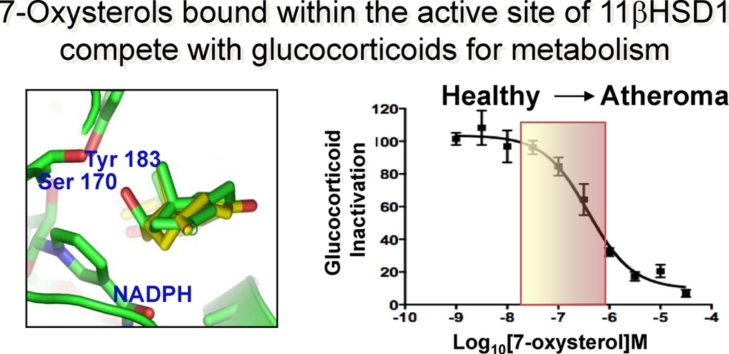

## 1 Introduction

Intracellular generation of active glucocorticoids (cortisol in humans, corticosterone in mice) is catalyzed by 11β-hydroxysteroid dehydrogenase (11βHSD) type 1 (EC 1.1.1.146). The potential for 11βHSD1 to regulate fuel metabolism has been demonstrated in murine models, in which disruption of the enzyme protects from metabolic dyshomeostasis [1,2] and, more recently, in humans in whom specific 11βHSD1 inhibitors improve hyperglycaemia [3]. In murine models, inhibition of 11βHSD1 also offers atheroprotection [4–6]. Therefore inhibition of the reductase activity of 11βHSD1 is a tractable target for drug development, but to fully understand the spectrum of actions and side-effects of such drugs, effects on other substrates of 11βHSD1 must be considered. This is, as yet, unexplored *in vivo*, either in genetically modified mice or following selective pharmacological manipulation.

**Fig. 1:**
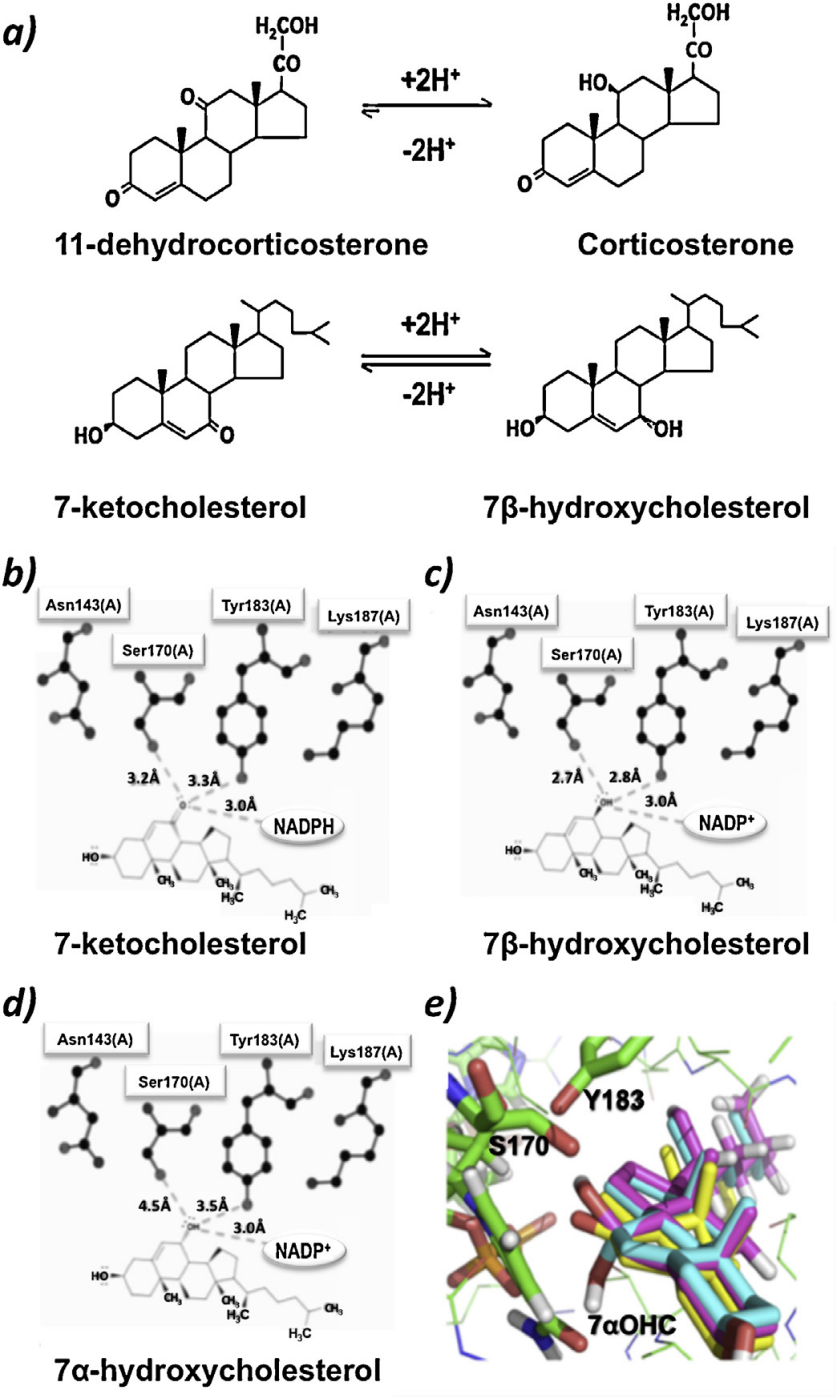
(a) Interconversion of glucocorticoids and 7-oxysterols catalyzed by 11β-hydroxysteroid dehydrogenase type 1 (11βHSD1). The equilibrium of interconversion of inert 11-keto and active 11β-hydroxy forms of glucocorticoids (shown here as 11-dehydrocorticosterone and corticosterone, the principle rodent glucocorticoids) favors predominant reduction. 11βHSD1 can also interconvert 7-keto and 7β-hydroxycholesterol but the favored equilibrium position between the two reactions is not understood. (b)–(e) *In Silico* modeling of interactions between 7-oxysterols and residues in the active site of murine 11β-hydroxysteroid dehydrogenase 1 (m11βHSD1). 2D Modeling of the active site of m11βHSD1 (retrieved from PDB 1Y5 M) using LigPlot. Hydrogen bond lengths of interactions between (b) 7-ketocholesterol and (c) 7β-hydroxycholesterol and the critical residues of catalytic tetrad are shorter than those for (d) 7α-hydroxycholesterol (7αOHC). (e) 3D modeling of interactions between active site residues Serine 170 (S170) and Tyrosine 183 (Y183) of m11βHSD1 and the 7-oxygenated moieties using PyMOL. Positioning of 7βOHC (pink) or 7KC (yellow) into the active site demonstrated their more favorable orientation over 7αOHC (turquoise), for hydrogen bonding with key amino acids of m11βHSD1 active site.

In addition to metabolizing glucocorticoids, 11βHSD1 can catalyze the inter-conversion of 7-keto- and 7β-hydroxy-sterols and steroids (Fig. 1a) (e.g. 7-oxygenated metabolites of dehydroepiandrosterone [7] and highly cytotoxic cholesterol metabolites, the 7-oxysterols [8,9]). 7-Oxysterols are formed from cholesterol both enzymatically and by auto-oxidation [10]. They accumulate in atherosclerotic plaques, a site of 11βHSD1 expression [11], with 7-ketocholesterol (7KC) being the most abundant, closely followed by 7β-hydroxycholesterol (7βOHC) [12]. Early reports [13,14] revealed that 11βHSD1 converted 7βOHC to 7KC in hepatic microsomes from all vertebrates tested (human, guinea-pig, rat, hamster and chicken) and that rat hepatic 11βHSD1 also reduced 7KC to 7βOHC. However, this has not been studied in other species and it remains unclear whether enzymes other than 11βHSD1 also catalyze interconversion of 7βOHC and 7KC.

11βHSD1 is a bi-directional enzyme (Fig. 1a) and both dehydrogenase (inactivating glucocorticoids) and reductase (regenerating glucocorticoids) activities can be measured in tissues [15,16]. The prevalent direction of 11βHSD1, with respect to metabolism of glucocorticoids, is reduction and is dependent on the availability of endogenous co-factor (NADPH), which is generated by hexose-6-phosphate dehydrogenase (H6PDH) within the endoplasmic reticulum (ER) [17]. Mice lacking H6PDH are unable to regenerate glucocorticoids by 11βHSD1 [18] but it is unclear if NADPH supply physiologically regulates the balance between reductase and dehydrogenase activities and the contribution of H6PDH *in vivo* has not been investigated for 7-oxysterols. Pharmacological inhibition of 11βHSD1 in rats caused hepatic accumulation of 7KC [9] suggesting that, as with glucocorticoids, the predominant direction of metabolism of 7-oxysterols by 11βHSD1 *in vivo* is reduction. Tissue-specific differences in the equilibrium position of metabolism of glucocorticoids by 11βHSD1 may indeed be due to the presence of competitive substrates, as some reports have suggested that 7-oxygenated compounds inhibit metabolism of glucocorticoids by 11βHSD1 [19]. For example, 7KC and 7βOHC inhibit 11βHSD1 activity in mouse adipocyte (3T3-L1 and 3T3-F442) cell lines [20] and in differentiated human THP-1 macrophages [21], modulating the downstream actions of glucocorticoids.

We hypothesized that 11βHSD1 is a key determinant of the balance of 7βOHC and 7KC *in vivo*. Depending on their levels in the circulation and tissues, 7KC and 7βOHC may differentially inhibit either reduction or dehydrogenation of glucocorticoids, respectively. Since these oxysterols accumulate in tissues that express 11βHSD1 [10] (e.g. macrophages, foam cells, adipose, atherosclerotic plaques [11]), the relative proportion of 7KC to 7βOHC may influence the amount of active glucocorticoid within cells. To address this hypothesis we investigated the balance of 7KC and 7βOHC in mice with transgenic disruption of 11βHSD1 and H6PDH, and abilities of these 7-oxysterols to influence the equilibrium between the dehydrogenase and reductase activities of glucocorticoid metabolism by murine 11βHSD1.

## 2 Materials and methods

Unless otherwise stated, solvents were HPLC grade (Fisher, Hemel Hempstead, UK) and contained an anti-oxidant (0.01%w/v butylated hydroxytoluene (BHT)). Steroids and oxysterols were from Steraloids (Newport, Rhode Island, USA), derivatization reagents from Fluka (Buchs, Switzerland), tissue culture reagents from Lonza (Reading, UK) and other chemicals from Sigma–Aldrich (Poole, UK). Tritiated 11-dehydrocorticosterone (11-DHC) was synthesized [22] from [1,2,6,7-^3^H]_4_-corticosterone (GE Healthcare, Bucks, UK). Deuterium-labeled internal standards [25,26,26,26,27,27,27-^2^H]_7_-7KC, [25,26,26,26,27,27,27- ^2^H]_7_-7βOHC and [25,26,26,26,27,27,27-^2^H]_7_-cholesterol were from CDN Isotopes (Essex, UK). Protein concentrations were quantified using a Bio-Rad kit (Hemel Hempstead, UK).

### 2.1 Animals

Male mice (10–16 weeks, n = 6–8/group [2,23]) with disruption of 11βHSD1 (*Hsd11b1*^−/−^) or H6PDH *(H6pdh*^*−/−*^*)* or both (*Hsd11b1*^*−/−*^*/H6pdh*^*−/−*^*)* and their wild-type littermate controls (15 weeks) were maintained on chow diet and tap water ad libitum, under a 16 h/8 h light/dark cycle at 21–24 °C. Male *ApoE*^*−/−*^ mice (in-house colony, 8 weeks; *n* = 6) were maintained on a western Diet (D12079B, Research Diets, USA) for 14 weeks. All licensed procedures were performed under accepted standards of humane animal care, as outlined in the UK Home Office Ethical Guidelines. Animals were culled by cervical dislocation at 10:00 h. Tissues and fluids were snap-frozen and stored at −80 °C.

### 2.2 Cell culture

HEK293 cells stably expressing full-length murine 11βHSD1 (m11βHSD1) [24] were maintained in Dulbecco's Modified Eagle Medium (DMEM) and seeded on poly-d-lysine coated (50 μg/mL, 5 min) plates. Medium was supplemented with glutamine (2 mM), penicillin (100 units/mL), streptomycin (100 μg/mL), and heat-inactivated fetal calf serum (10%v/v). For assessment of kinetic parameters, stripped fetal calf serum, prepared with dextran-coated charcoal (1% w/v), was added to the cells 12 h prior to use. For manipulation of cholesterol, medium was replaced with serum free medium 1 h prior to experimentation. Cells were maintained in a humidified atmosphere (5%CO_2_, 95% air, 37 °C).

### 2.3 Quantitation of 11βHSD1 enzyme kinetics

Inter-conversion of substrates and products was quantified under conditions of first order kinetics. Three forms of murine enzyme (*n* = 6/group) were used: (1) a truncated form of recombinant m11βHSD1 protein (N23Δ, gift from Dr Webster), (2) enzyme contained within murine hepatic microsomes and (3) a full-length m11βHSD1 protein expressed in stably transfected HEK293 cells [24].

#### 2.3.1 Metabolism by purified and microsomal murine 11βHSD1

Recombinant (14–28 μg/mL) or murine hepatic microsomal (240–260 μg/mL [19]) protein was incubated (30 min, 37 °C) with 7-oxysterols (0.02–20 μM) in potassium phosphate buffer (0.1 M, 0.1 mM EDTA, 20 mM cysteamine hydrochloride, pH 7.4), or with steroids (0.02–20 μM) in Krebs-Ringer buffer (containing 5 mM glucose), and the relevant cofactor (2 mM) for oxidation (NADP+ or NAD+) or reduction (NADPH or NADH). Similar experiments were performed in hepatic microsomes from *Hsd11b1*^−/−^ mice [2] (substrate 0.2 μM) with and without induction of cationic permeability by alameticine (0.25 mM, 1–2 h). Reactions did not proceed in the absence of either protein or the co-factor.

Following incubation, internal standards (epi-cortisol for steroids or 19-hydroxycholesterol (19OHC, 100 ng) and 4α-cholesten-7α-ol-3-one (50 ng) for oxysterols) were added after stopping the reaction with addition of ethyl acetate (steroids; 10 vol) or petroleum ether (oxysterols; 10 vol). Organic extracts were reduced to dryness under oxygen free nitrogen (60 °C) or argon (room temperature), respectively, and the residues stored at −20 °C until analysis by HPLC.

#### 2.3.2 Metabolism by recombinant murine 11βHSD1 expressed in stably transfected cells

HEK293 cells, stably transfected to produce m11βHSD1, were seeded onto a 5 cm dish and incubated overnight with 7KC, 7βOHC or 7αOHC (1 μM), or with steroid (30 nM) for 45 min. Following addition of internal standard, as above, oxysterols were extracted from the medium into 2-propanol:hexane (40:60, 9 mL, 50 μg/mL BHT) [25]. Dried organic residues were stored at −20 °C until analysis by gas chromatography mass spectrometry (GCMS). Reactions did not proceed in non-transfected HEK293 cells.

### 2.4 Competition between 7-oxysterols and glucocorticoids for metabolism by 11βHSD1

Recombinant protein (20 μg/mL) was incubated, as above, in the presence of 11-DHC (0.5–10 μM) or corticosterone (0.025–0.2 μM) and 7KC (0.02–20 μM) or 7βOHC (0.02–10 μM). Murine hepatic microsomes (260 μg/mL) were incubated, as above, with steroid (0.2 μM) in the presence of 7βOHC or 7KC (0.1 nM-5 μM). The velocity of metabolism of steroids (0.02–5 μM) was further assessed in the presence of 7-oxysterols at their IC_50_ concentration (vehicle, 0.01% *v/v* ethanol). HEK293 cells expressing m11βHSD1 were cultured, as above, and incubated with [^3^H]_4_-labeled (5 nM) and unlabelled (25 nM) steroid and 7βOHC or 7KC or other oxysterols (7α-, 19-, 22R- or 27-OHC; 1 nM-5 μM, 0.01% v/v ethanol control).

### 2.5 Supplementation of cholesterol in stably transfected cells

To enrich cellular cholesterol and 7-oxysterol content, HEK293 cells stably expressing m11βHSD1 were incubated (37 °C, 30 min) with cholesterol-loaded methyl-β-cyclodextrin (1:6, 10 mM in DMEM) [26]) and kinetic experiments performed within 24 h. Following manipulation, cells were washed with DMEM (37 °C) followed by phosphate buffered saline, and then incubated (5 × 10^6^ cells/well, 1 h) in serum-free DMEM containing either [^3^H]_4_-corticosterone or [^3^H]_4_-dehydrocorticosterone (30 nM). Products of metabolism were quantified in medium by HPLC. Following incubation, cells were washed with ice-cold PBS and then lysed by gently rocking with NaOH (200 μM, 0.6 mL/well, 15 min, 4 °C) [27]. An aliquot of lysate was retained for quantitation of protein. To the remaining cellular lysate, internal standards [^2^H]_7_-7KC, [^2^H]_7_-7βOHC (50 ng) and [^2^H]_7_-cholesterol (1 μg) were added and oxysterols and cholesterol were immediately extracted into methanol:hexane (2:5, 7 mL, 50 μg/mL BHT, 2 mM EDTA). The dried organic extract was dissolved in chloroform: methanol (2:1) and processed for quantitation by GCMS. All final measurements were expressed as a ratio of the total protein content in the cells.

### 2.6 Quantitation of circulating and tissue levels of 7-oxysterols

7-Oxysterols were quantified in plasma (0.4–1 mL) prepared from trunk blood collected (pooled if necessary) in EDTA-coated tubes from mice (*n* = 8/group). Plasma was prepared from blood collected in EDTA-coated (1.6 mg/mL) vials. The effects of disruption of *Hsd1b1*, *H6pdh* or both were explored in hepatic microsomes and cytosol (0.05–0.5 mg/mL protein) from mice homozygous for the disrupted allele (*n* = 6/group) versus their littermate controls. All samples were flushed under argon prior to extraction and BHT (45 mM, in ethanol) added before 7-oxysterols were extracted and converted to their trimethylsilyl derivatives [28] prior to analysis by GCMS [29].

### 2.7 Quantitation of steroids and oxysterols by HPLC

Substrates and products in *in vitro* extracts were analyzed by HPLC (Dionex SUMMIT^®^ system, Camberley UK) with online radioscintillation detection (LB509^®^ β-scintillation counter, Berthold Technologies GmbH & Co, Germany). 7-Oxysterols were eluted from a SUNFIRE^®^ column (C18, 15 cm, 4.6 mm, 5 μm; Waters, Edinburgh, UK) with acetonitrile:water (95:5), at 1 mL/min, 24 °C and quantified at selected wavelengths (195 nm (7KC, 4α-cholesten-7α-ol-3-one), 237 nm (hydroxycholesterols)). Glucocorticoids were separated using a SYMMETRY^®^ C8 column maintained at 35 °C (15 cm, 4.6 mm, 5 μm, Waters) using a mobile phase of water:acetonitrile:methanol (60:15:25) flowing at 1 mL/min. Unlabelled steroids were detected at 240 nm. Unlabelled oxysterols and steroids were quantified by interpolation onto a standard curve of peak area divided by that of the internal standard vs concentration, prepared from calibration standards processed simultaneously. Abundances of tritiated steroids were quantified by on-line liquid scintillation counting (2 mL/min; GOLDFLOW^®^, Meridian, Surrey, UK).

### 2.8 In silico modeling of interactions between 7-oxysterols and residues in the active site of murine 11β-hydroxysteroid dehydrogenase 1 (m11βHSD1)

3D Macromolecular structural information about m11βHSD1 was obtained from the Research Laboratory for Structural Bioinformatics Protein Data Bank. 1Y5 M represented a dimeric m11βHSD1 bound with NADP+ and 1Y5R represented m11βHSD1 bound with NADP+ and corticosterone [30]. The structure of 7α-hydroxysteroid dehydrogenase (EC1.1.1.159, 7αHSD, PDBID 1FMC) in complex with 7-oxoglycochenodeoxycholic acid [31] was a template for modeling the steric orientation of 7αOHC, allowing alignment of 7α- and 7β-hydroxyl and 7-keto groups into the active site, when 7αHSD and 11βHSD1 were subsequently superimposed. Energy maps for all ligand atoms around the active site were generated using the virtual screening program LIDAEUS (Ligand Discovery At Edinburgh University). Energy minimization routines were used to aid the positioning of substrate within the active site of 11βHSD1. 2D Representations of protein-ligand complexes from modeled structures were created using LigPlot (Cambridge, UK), the output of which was then augmented by 2D representations of substrates generated by MARVINVIEW^®^ (ChemAxon, Budapest, Hungary) to distinguish between the steric orientation of 7α- and 7βOHC. Visualization of 3D structures was performed using PyMOL (open source, DeLano Scientific LLC).

### 2.9 Analysis of kinetics and statistics

*V*_max_, *K*_m_ and *K*_*i*_ values were determined, using global nonlinear regression, from data generated by measuring reaction velocity across a range of substrate concentrations in the absence and presence of competitor. In addition, using recombinant protein, Dixon Plots were generated as the reciprocal of the reaction velocity using four substrate concentrations [*S*] against four inhibitor concentrations [*I*]. All data are presented as mean ± SEM. Non-linear regression and statistical comparisons were made using GRAPHPAD PRISM^®^ software v5.0 (GraphPad Software Inc. San Diego, USA) by 1 or 2-way ANOVA (with Tukey post hoc tests), or unpaired or paired Student's *t*-tests as appropriate.

## 3 Results

### 3.1 Disruption of 11βHSD1 or H6PDH *in vivo* impairs reduction of 7-oxysterols

7-Oxysterols were present in plasma from wild-type, littermate control mice in concentrations of 91.3 ± 22.3 (7KC) and 22.6 ± 5.7 (7βOHC) nM [29]. Levels increased more than 10 fold (1240 ± 22 (7KC) and 406 ± 39 (7βOHC) nM) in *ApoE*^−/−^ mice on an atherogenic, western diet. Following disruption of *Hsd11b1*, there was a trend (*p* = 0.08) for an increase in concentrations of 7KC (133.8 ± 16.8 nM) but not 7βOHC (23.6 ± 2.2 nM) [29]. However, the ratio of 7KC/7βOHC in plasma significantly increased in the *Hsd11b1*^−/−^ (5.4 ± 0.5) vs. control mice (4.1 ± 0.4, *n* = 9, *p* < 0.05).

Both 7KC and 7βOHC were detected in microsomes from control mice. Disruption of *Hsd11b1* caused a profound reduction in hepatic microsomal concentrations of both oxysterols (Table 1), with an increase in the 7KC/7βOHC ratio. In the cytosols from control murine liver, only 7KC (25.3 ± 13.4 ng/mg protein) was detected, but following disruption of 11βHSD1, levels of 7KC became undetectable. Disruption of *H6pdh*, or both *H6pdh* and *Hsd1b1* also lowered the levels of 7βOHC and 7KC in the hepatic microsomes compared with littermate controls (Table 1). The 7KC/7βOHC ratio increased with disruption of *H6pdh* and disruption of both *H6pdh* and *Hsd11b1* did not have any further effect over lack of 11βHSD1 alone (Table 1).

#### 3.1.1 Oxysterols Inhibit oxidation and/or reduction of glucocorticoids

Competition between 7-oxysterols and glucocorticoids for metabolism by 11βHSD1 across physiological and pathophysiological concentration ranges was investigated using three preparations of murine enzyme. In all preparations, 7αOHC was not accepted as a substrate and not generated upon reduction of 7KC (not shown).

#### 3.1.2 Murine 11βHSD1 stably transfected into HEK293 cells

**Fig. 2:**
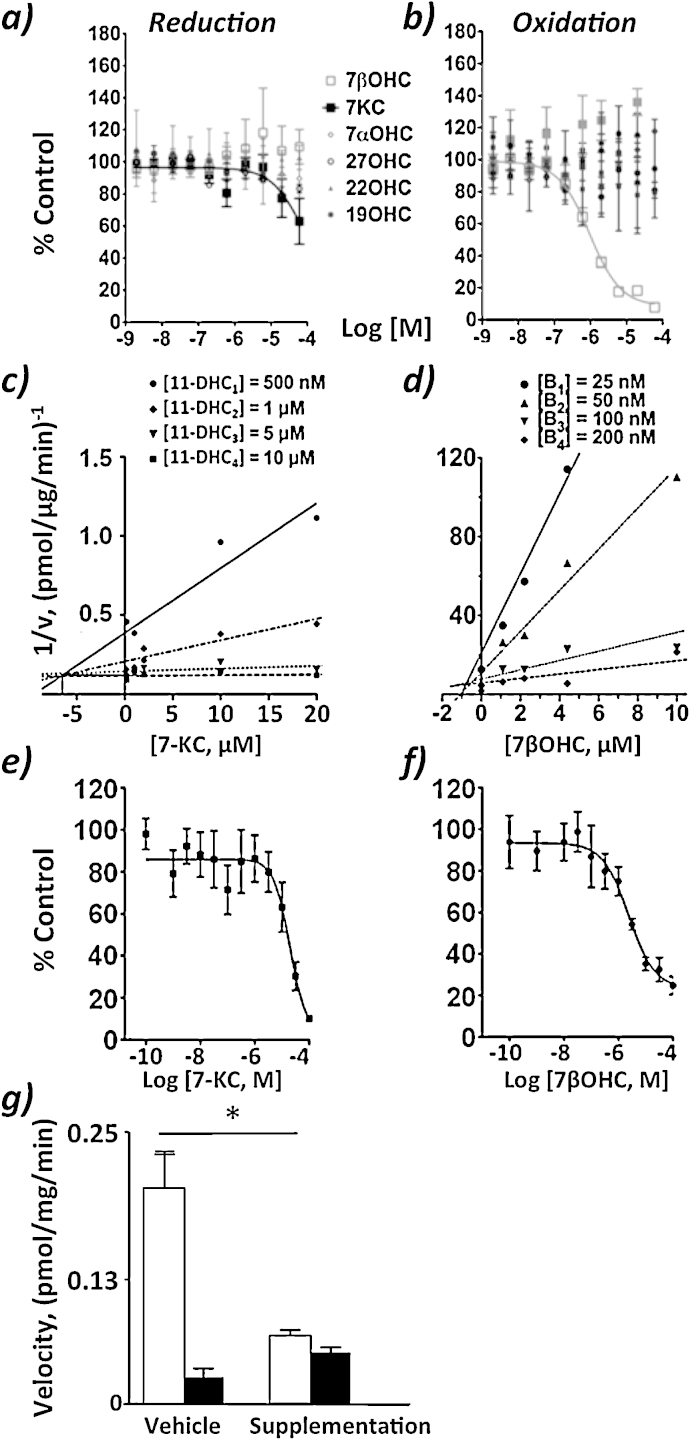
7-Oxysterols inhibit the metabolism of glucocorticoids by 11β-hydroxysteroid dehydrogenase 1 (11βHSD1). (a) and (b): The velocities of (a) reduction of 11-dehydrocorticosterone (11-DHC) to corticosterone and (b) oxidation of corticosterone to 11-DHC were quantified following incubation of HEK293 cells (stably transfected to generate murine 11βHSD 1) with a range of concentrations of oxysterols. Non-linear regression was used to assign IC_50_ values. 7-Ketocholesterol (7KC) only inhibited the reduction of 11-DHC by ∼40%. 7β-Hydroxycholesterol (7βOHC) completely inhibited oxidation of corticosterone; other oxysterols did not have an effect. Data (mean ± SEM) are % control (absence of oxysterol), *n* = 6 for 7-oxysterols and *n* = 3 for other oxysterols. OHC = hydroxycholesterol. (c)–(f) 7-Oxysterols inhibited metabolism of glucocorticoids by recombinant and microsomal 11βHSD1; in both cases they inhibited dehydrogenation more potently than reduction. Competitive inhibition of (c) reduction of 11-DHC to corticosterone in the presence of 7KC and (d) oxidation of corticosterone to 11-DHC in the presence of 7βOHC, by recombinant 11βHSD1, demonstrated by Dixon Plots (mean data). Inhibition of (e) reduction of 11-DHC to corticosterone in the presence of 7KC and (f) oxidation of corticosterone to 11-DHC in the presence of 7βOHC by microsomal 11βHSD1. *n* = 3–7. (g) Supplementation of cholesterol impeded reduction of glucocorticoids by 11βHSD1. The velocity of reduction of 11-DHC (open bars) by 11βHSD1 stably transfected in HEK293 was suppressed when 7-oxysterol levels were supplemented by delivery of a complex of cholesterol and methyl-β-cyclodextrin (1:6). *p < 0.01 compared by 2 way ANOVA with Bonferroni post-test, *n* = 6.

Both oxidation and reduction of glucocorticoids were detected *in vitro*, and reduction was the preferred direction (0.79 ± 0.15 (oxidation) vs. 3.86 ± 0.27 (reduction) pmol/mg/min, respectively, with 30 nM substrate). Both oxidation of 7βOHC and reduction of 7KC, were observed, at similar velocities, which were considerably slower than those measured for glucocorticoids. For example, substrate concentrations of 1 μM were required to achieve rates of oxidation of 7βOHC and reduction of 7KC of 0.90 ± 0.31 vs 0.74 ± 0.04 pmol/mg/min, respectively. Inhibition of metabolism of glucocorticoids by a range of endogenous oxysterols was assessed in both reductase and dehydrogenase directions. 7KC caused the most marked inhibition of reduction of all oxysterols tested, although still only by 40% at the highest concentration used (100 μM; Fig. 2a) and further kinetic analysis was not performed. Of the different oxysterols tested, only 7βOHC inhibited oxidation of corticosterone, with a *K*_*i*_ of 1.77 ± 0.09 μM (Fig. 2b).

#### 3.1.3 Murine recombinant 11βHSD1

Although both oxidation and reduction of glucocorticoids were detected using recombinant 11βHSD1, reduction of 11-DHC was the favored reaction (lower *K*_m_ and higher *V*_max_, Table 2). Oxidation of 7βOHC and reduction of 7KC were also detected but proceeded with slower maximal rates and these substrates had poorer affinity (higher *K*_m_s; Table 2) than glucocorticoids. 7KC inhibited reduction of 11-DHC (Fig. 2c) with a *K*_*i*_ of 7.33 ± 1.76 μM, and 7βOHC inhibited dehydrogenation of corticosterone with a *K*_*i*_ of 0.91 ± 0.05 μM. (Fig. 2d). In both cases, the nature of inhibition was competitive, indicated by the regression lines of the Dixon Plots intercepting above the *x*-axis.

#### 3.1.4 11βHSD1 in murine hepatic microsomes

Both oxidation and reduction of glucocorticoids were detected using microsomal 11βHSD1 with reduction being the preferred direction (lower *K*_m_ and higher *V*_max_, Table 2). In contrast, only oxidation of 7βOHC could be measured, forming 7KC at the same rate in the presence of either NAD+ or NADP+ (e.g. 1.25 ± 0.2 vs. 1.35 ± 0.4 pmol/μg/min respectively; 20 μM substrate, *n* = 3). This reaction was dependent on the presence of 11βHSD1, as 7βOHC was not converted to 7KC by hepatic microsomes from *Hsd11b1*^−/−^ mice, with either cofactor. Again, 7-oxysterols demonstrated poorer affinity than glucocorticoids for 11βHSD1. The *K*_m_ for oxidation of 7βOHC was approximately three orders of magnitude higher than that for glucocorticoids (Table 2), although the maximal velocities achieved were similar for glucocorticoids and 7-oxysterols. Reduction of 7KC could not be demonstrated, even following the addition of the permeabilisation agent, alameticine, or use of NADH as an alternative cofactor [32]. 7KC weakly inhibited reduction of 11-DHC with an IC_50_ of 19.4 ± 1.2 μM (Fig. 2e) and further kinetic analysis was not performed. 7βOHC inhibited oxidation with an IC_50_ of 2.2 ± 0.4 μM (Fig. 2f).

### 3.2 Supplementation of cellular content of cholesterol and 7-oxysterol impedes reduction of glucocorticoids by 11βHSD1

The effect of cholesterol loading was assessed on the equilibrium of 11βHSD1 stably transfected into HEK293 cells. 7KC (19.4 ± 1.08 pmol/mg) and 7βOHC (4.37 ± 1.90 pmol/mg) were present in cells treated with vehicle. Cholesterol loading significantly (*p* < 0.05) increased the levels of 7KC (39.48 ± 3.01 pmol/mg) and 7βOHC (17.6 ± 2.4 pmol/mg), associated with a slower velocity of reduction of glucocorticoids by 11βHSD1 compared with vehicle-treated cells (Fig. 2g).

### 3.3 In silico modeling

#### 3.3.1 2D modeling

A representation of m11βHSD1 (PDB structure 1Y5R) was created to predict proximity of interactions of steroids and 7-oxysterols with resident cofactor (NADP+/NADPH) and the tyrosine (Tyr183) and serine (Ser170) residues of the catalytic tetrad (Tyr183-Ser170-Lys187-Asn143) (Figs. 1(b)–(d)) [33]. Distances of hydrogen bonds from the active C7 oxygen on 7-oxysterols to Ser170 and Tyr183 residues were shortest for 7βOHC (2.7, 2.8 Å respectively), longer for 7KC (3.2, 3.3 Å) and longest for 7αOHC (4.5, 3.5 Å). Distances of 7βOHC and 7KC were comparable to those of glucocorticoids (B, 3.0, 3.2; A, 2.8, 2.6 Å respectively). Interactions with the co-factor were similar for all three 7-oxysterols (all 3.0 Å; corticosterone 3.9, 11-DHC, 3.3 Å).

#### 3.3.2 3D in silico modeling

7-Oxysterols have not been co-crystallised with 11βHSD1. Thus, to establish the spatial orientation of the oxygenated residues at the C7 position, the structure of the closely related 7αHSD in complex with 7-oxoglycochenodeoxycholic acid (1FMC) was used. Tyrosine residues in the active sites of 7αHSD (1FMC) and m11βHSD1 (1Y5R) could be superimposed, allowing the 7α-hydroxyl group of 1FMC ligand to overlay the 11β-hydroxyl group of corticosterone docked within 1Y5R. Thus, the 3D structure of 7αOHC was created to resemble that of 7-oxoglycochenodeoxycholic acid, allowing the positions of the 7β-hydroxyl and 7-keto groups of 7βOHC and 7KC respectively to be orientated. 7-Oxysterols were docked into the active site of 1Y5R and 3D representations shown in Fig. 1e. The A-ring of 7-oxysterols (as opposed to the D-ring of glucocorticoids) was orientated toward the interior of the 11βHSD1 active site. Interactions between 7βOHC and Ser170 and Tyr183 of the catalytic tetrad had the shortest bond distances (2.7, 2.8 Å respectively), followed by those of 7KC (3.2, 3.3 Å) and then 7αOHC (4.5, 4.8 Å; for comparison corticosterone 3.1, 2.8 Å [30]; 11-DHC 2.8, 2.6 Å respectively) When 7βOHC and 7KC were docked, the Tyr183 residue was 5.1 Å from the nicotinamide C4 and Lys187 was 3.2 Å from the hydroxyl group on the cofactor. When 7αOHC was docked, the Tyr183 residue was 4.20 Å from the nicotinamide C4 and Lys187 was 3.2 Å from the hydroxyl group on the cofactor.

## 4 Discussion

These data demonstrate that reduction of 7KC to 7βOHC is the preferred direction of metabolism of 7-oxtserols by 11βHSD1 *in vivo* in mouse. Metabolism of 7-oxysterols (at least dehydrogenation) was not detected in microsomes of 11βHSD1 null mice, supporting the notion that it is the only enzyme catalyzing this reaction. While 7-oxysterols were competitive inhibitors of metabolism of glucocorticoids by 11βHSD1, it is unlikely that in health [34] they will be sufficiently potent to exert this effect. Inhibition may become important in hyperlipidaemia [10], or at sites where oxysterols accumulate, such as in adipose and atherosclerotic lesions.

Structural modeling of the murine protein confirmed the potential for interactions of 7-oxysterols with the catalytic tetrad of the enzymatic active site. 7-Oxygenated substrates, in contrast to steroids, interact with 11βHSD1 with their A-ring orientated toward the interior of the binding pocket, in agreement with models in other species [21,30,35]. The higher *K*_m_ values describing metabolism of 7-oxysterols compared with glucocorticoids, however, indicated they were poorer affinity substrates. Circulating concentrations of 7-oxysterols in the mouse were comparable in magnitude to those in other species [34] and increased in hyperlipidaemia [10]. However, it is likely metabolism by 11βHSD1 would not proceed at maximal velocity in the presence of the endogenous concentrations reported here or by others [34,36].

While disruption of 11βHSD1 only tended to alter circulating 7-oxysterol levels subtly [29], it substantially lowered the levels in hepatic sub-cellular fractions. Oxysterols can be synthesized from spontaneous oxidation of cholesterol and are derived in large part from dietary sources [10]. Therefore the reduction in absolute levels may arise because *Hsd11b1*^*−/−*^ mice have an improved metabolic profile with lower circulating cholesterol concentrations [37], and thus less precursor for auto-oxidation. The specific contribution of 11βHSD1 to the proportions of 7-keto and hydroxy oxysterols was revealed in the increase in the ratio of 7KC/7βOHC ratio in plasma and microsomes, following targeted disruption of *Hsd11b1*, suggesting that 11βHSD1 catalyses reduction of 7KC *in vivo*. This corroborates previous studies in rats in which hepatic 7KC accumulated following administration of the non-specific 11βHSD inhibitor carbenoxolone [9]. Lack of NADPH supply due to genetic disruption of *H6pdh* again increased the 7KC/7βOHC ratio, confirming *in vitro* findings [38] that H6PDH promotes catalysis of 7KC to form 7βOHC *in vivo*, similarly to glucocorticoids. Indeed, H6PDH appeared to be the only source of co-factor, as double knockout of *H6pdh* and *Hsd11b1*, yielded the same ratio of 7-oxysterols, as with disruption of *H6pdh* alone.

11βHSD1 may therefore play a similar role in regulating actions of 7-oxysterols *in vivo* as it does glucocorticoids. The importance of metabolism of glucocorticoids by 11βHSD1 is readily apparent since the 11-keto steroid is inert and the hydroxy form is active. However, distinct biological roles for 7KC and 7βOHC are not established and a target receptor has not been defined, although there are a number of reports of subtle differences in their actions (e.g. 7βOHC has a greater ability than 7KC to induce apoptosis in human umbilical vein endothelial cells [39]). However 7-oxysterols can be subject to further metabolism and recent reports suggest that the 25- and 27-hydroxy metabolites of 7α- and 7βOHC play potential roles in regulating the immune response via the novel G-protein coupled receptor, EB12 [40,41]. Interestingly there is one report showing that 7KC but not 7βOHC limits SCAP exit from the ER within cells [42], which further prevents excess synthesis of cholesterol. Hence, it follows that the increased proportion of 7KC to 7βOHC upon inhibition of 11βHSD1 *in vivo* may exert a brake on cholesterol synthesis. Other oxysterols modulate nuclear hormone signaling pathways, but the possibility of activation of LXR, at least, by 7-oxysterols has largely dismissed [20].

Work with cells stably transfected to express human 11βHSD1 or with adipocytes [20] has shown that 7-oxysterols (in keeping with other 7-hydroxylated substrates [7]) may compete differentially with glucocorticoids for metabolism by 11βHSD1 and thus modulate glucocorticoid action. Inhibition appears cell-type specific, potentially explained by differential metabolism, accumulation or export of oxysterols [10]; adipocytes and macrophages sequester oxysterols readily [10] whereas macrophages export 7KC and other oxysterols via the ABCG1 transporter [43]. Balázs et al. did not detect any inhibition of human 11βHSD1 reductase activity in lysates or HEK293 cells by 7KC or 7βOHC, but showed an inhibition of 11βHSD1-reductase activity by 7KC (IC_50_ 8.1 ± 0.9 μM) in differentiated THP-1 macrophages [21]. Inhibition of glucocorticoid metabolism by co-incubation with 7-oxysterols was investigated here using three models of murine 11βHSD1, in all of which reduction of glucocorticoids was favored. Our data concur with the proposal that 7-oxysterols compete with glucocorticoids for metabolism, with 7KC being consistently less effective at inhibiting 11-DHC reduction by isolated enzyme *in vitro*, than 7βOHC was at preventing oxidation. Taking into account the IC_50_ values, inhibition of glucocorticoid metabolism is unlikely to be important in health. However, at concentrations in the low micromolar range, as seen in atherosclerosis [44,45], 7βOHC or 7KC may compete for oxidation preventing glucocorticoid inactivation or reduction, respectively.

7βOHC is highly abundant in fatty streaks in developing lesions [46,47] and the 7βOH/7KC ratio is increased. If 7βOHC dominates to inhibit glucocorticoid oxidation, the cells in the lesion and adjacent normal intima may become exposed to increased local glucocorticoid levels, with adverse consequences [48]. However, when endogenous 7-oxysterols were enriched secondary to cholesterol loading in cultured cells, the predominant effect was to suppress reduction of glucocorticoids, suggesting protection from excess glucocorticoid. These findings concur with reduction of 7KC being the major route of metabolism of 7-oxysterols *in vivo*.

In conclusion, 7KC and 7βOHC are poor affinity substrates for murine 11βHSD1 and are interconverted at a slower rate than glucocorticoids. While differences exist in the patterns of *in vitro* and *in vivo* metabolism, reduction of 7KC to 7βOHC appears the predominant reaction *in vivo*. Although it seems unlikely that the competition with oxysterols will determine predominant direction for glucocorticoid metabolism by 11βHSD1 in health, it may play a role in hyperlipidaemia and atherosclerosis. A greater knowledge of the actions of these 7-oxysterols is required to fully understand the consequences of inhibition or over-activity of 11βHSD1 pathway.

## Figures and Tables

**Table 1:** Effect of disruption of *Hsd1b1* or *H6pdh* on 7-oxysterol concentrations in hepatic microsomes.

	Control	Hsd11b1^−/−^	H6pdh^−/−^	Hsd11b1^−/−^/H6pdh^−/−^
7βOHC	84.45 ± 27.55	16.10 ± 5.20**	12.12 ± 2.34**	21.44 ± 3.50*^,^#
7KC	22.45 ± 8.20	10.01 ± 2.85*	7.78 ± 1.68*	14.76 ± 2.64*^,^#
7KC/7βOHC	0.31 ± 0.14	0.65 ± 0.04*	0.63 ± 0.01*	0.68 ± 0.04**

Disruption of either 11β-hydroxysteroid dehydrogenase 1 (*Hsd11b1*^−/−^) or hexose-6-phoshate dehydrogenase (*H6pdh*^−/−^) reduced levels (ng/mg protein) of 7β-hydroxycholesterol (7βOHC) to a greater extent than 7-ketocholesterol (7KC) in hepatic microsomes compared with littermate control (C57BL/6) mice. Disruption of both enzymes (*Hsd11b1*^−/−^, *H6pdh*^−/−^) did not have any additional effect on the levels of 7-oxysterols over lack of 11βHSD1 or H6PDH alone. Data are mean ± SEM, compared using 1-way ANOVA, and Tukey's post hoc test, *n* = 4–6.

* *p* < 0.05.

** *p* < 0.01 vs. control.

# *p* < 0.05 compared with *H6pdh*^−/−^.

**Table 2:** Kinetic parameters describing metabolism of 7-oxysterols and glucocorticoids by murine 11β-hydroxysteroid dehydrogenase 1 (11βHSD1).

Substrate		*K*_m_ (μM)	*V*_max_	*V*_max_/*K*_m_
**Recombinant protein**				
11-Dehydrocorticosterone	Reduction	0.20 ± 0.25	8.56 ± 4.06	42.8
7-Ketocholesterol	Reduction	1 269 ± 282	0.12 ± 0.03	9 × 10^−5^
Corticosterone	Oxidation	1.78 ± 0.56	4.82 ± 0.65	2.7
7β-hydroxycholesterol	Oxidation	327.60 ± 98.50	0.010 ± 0.001	3 × 10^−5^
**Microsomes**				
11-Dehydrocorticosterone	Reduction	1.30 ± 0.54	1.19 ± 0.18	0.9
7-Ketocholesterol	Reduction	Product not detected		
Corticosterone	Oxidation	4.20 ± 2.01	0.04 ± 0.01	0.01
7β-hydroxycholesterol	Oxidation	3 500 ± 326	0.03 ± 0.001	9 × 10^−6^

Velocities of metabolism of substrates by murine recombinant or microsomal 11βHSD1 were assessed and kinetic parameters (*K*_m_, *V*_max_ and *V*_max_/*K*_m_, true or apparent) assigned following Lineweaver-Burke transformation of data fitted to Michaelis-Menten kinetics. The velocities were quantified; for reduction of 11-dehydrocorticosterone or 7-ketocholesterol in the presence of NADPH or oxidation of corticosterone or 7β-hydroxycholesterol in the presence of NADP^+^. Data are mean ± SEM, obtained from at least three independent experiments. *V*_max_ expressed as pmol/μg/min. *V*_max_/*K*_m_ expressed as L/μg/min × 10^−6^.
